# Effect of Acupuncture on Diaphragm Function in Healthy Volunteers: A Pilot Clinical Study

**DOI:** 10.1155/2022/6608200

**Published:** 2022-05-29

**Authors:** Paolo Formenti, Andrea Galimberti, Riccardo Pinciroli, Michele Umbrello

**Affiliations:** ^1^SC Anestesia e Rianimazione I, Ospedale San Paolo, ASST Santi Paolo e Carlo–Polo Universitario, Milano, Italy; ^2^U.O.C. Anestesia Rianimazione e Terapia Intensiva, ASST Nord Milano, Milano, Italy; ^3^Department of Anesthesia, Critical Care and Pain Medicine, Beth Israel Deaconess Medical Center, Harvard Medical School, Boston, MA, USA; ^4^SC Anestesia e Rianimazione II, Ospedale San Carlo Borromeo, ASST Santi Paolo e Carlo—Polo Universitario, Milano, Italy

## Abstract

**Introduction:**

In western medicine, the diaphragm is the main inspiratory muscle. It is involved in the postural control of the trunk and contributes to balance performance. In traditional Chinese medicine (TCM), the diaphragm represents a gateway between the lower and upper parts of the body, and it regulates the descending and ascending functions of the whole organism. The aim of this study was to assess the effect of acupuncture on diaphragm function.

**Methods:**

A proof-of-concept, prospective, controlled, pilot trial in healthy volunteers. Respiratory rate, inspired volume, diaphragm excursion, and thickening were measured during tidal and maximal breathing at baseline and after sham or real acupuncture. Acupuncture was performed on Yanglingquan (GB34), Tai Chong (LV3), Danzhong (CV17), Geshu (BL17), and Geguan (BL46).

**Results:**

Ten participants were enrolled (6 males, weight 71 ± 12 kg, height 173 ± 9 cm, BMI 21 ± 1.3 kg/m^2^). During tidal breathing, tidal volume, diaphragm displacement, and end-expiratory diaphragm thickness did not change with sham or real acupuncture as compared to baseline; thickening ratio was 43.5 ± 16.6 vs. 31.3 ± 14.9 (sham) vs. 30.8 ± 15.3% (baseline), *p*=0.1066. During breaths at vital capacity, the acupuncture group, as compared to both baseline and sham acupuncture, had a trend toward a larger tidal volume (3840 ± 690 vs. 3110 ± 880 vs. 3230 ± 750 ml, *p*=0.1247) and showed a significantly higher thickening ratio (270.6 ± 136.4 vs. 172.4 ± 57.4 vs. 188.6 ± 41.7%, *p*=0.0414).

**Conclusions:**

Acupuncture significantly influenced diaphragm function in healthy volunteers, improving its inspiratory thickening fraction during breaths at vital capacity, as compared to no or sham acupuncture.

## 1. Introduction

The diaphragm is a thin, dome-shaped muscle which separates the thoracic from the abdominal cavity. In western medicine, it is considered the most important inspiratory muscle, and its contraction leads to the expansion of the ribcage, reduction of intrathoracic pressure, and the consequent increase in lung volume [1]. Moreover, it contributes with other respiratory muscles (such as intercostals) to balance performance [2] and postural control of the trunk when the limbs move [3]. In traditional Chinese medicine (TCM), the role of the diaphragm is to separate the “upper” from the “lower” and the “inside” from the “outside”; moreover, as it separates the thorax from the abdomen, it allows the “pure” to flow toward the lungs, the heart, and the brain, while it prevents the “impure” to flow. According to TCM, the human body possesses three energy centers, known as the three Dantians (elixir or cinnabar field). Each Dantian is separated from the others by a diaphragm, or door, which allows them to interact [4]. Within TCM, at least two different “diaphragms” can be identified, respectively, by the ideograms Ge and Huang: in the context of the Yin-Yang dialectics, the former can be identified as the Yin, while the latter as the Yang. Although both are usually translated as “diaphragms,” Ge indicates the diaphragm as it is meant in the western culture, while Huang refers to the thoracic space. One of the first discussions about the origin and function of the diaphragm is reported in the chapter of the Ling Shu which deals with organ control points, in which a close relationship is suggested between the diaphragm and the organs [5]. Moreover, the diaphragm is strictly connected with the main meridians, a system of channels through which vital energy, or Qi, flows [6]. In fact, all of them cross the diaphragm and energy flows through it. Therefore, a disorder of the diaphragm can alter the functioning of any major organ (such as the heart, the liver, the spleen, the lung, and the kidney) and the diaphragm plays an essential role in the equilibrium of the energy that flows between the ascending and the descending. Eventually, the emotional function of the diaphragm has a primary importance in TCM: the organ works as an emotional shield and a bridge between conscious and unconscious emotions; it can lock the emotions and stuck them in the abdomen or make them flow upwards and alter the shen [7]. In summary, the diaphragm represents a gateway between the lower and upper parts of the body, as it regulates the descending and ascending functions of the whole organism [8]. As opposed to TCM, in western medicine, the main role of the diaphragm is that of an inspiratory muscle. It is innervated by the phrenic nerve (which is formed in the neck within the cervical plexus and contains fibres from spinal roots C3–C5) and is composed of type I (slow and resisting to prolonged effort) and type IIa muscle fibres (fast and strong in the short period). Its dome shape, together with the anatomical relations with thoraco-abdominal structures, makes this organ essential for an effective breathing [9]. Mechanical ventilation is one of the most common forms of organ support needed by critically ill patients admitted to an Intensive Care Unit [10]. The temporary need to take over the patient respiratory function in case of respiratory failure might lead to several undesired effects, among which a relevant role is played by diaphragm dysfunction [11–14]. In fact, an excessive unloading of the diaphragm may lead to the development of disuse atrophy, while an insufficient ventilator assistance may be associated vigorous spontaneous efforts and the consequent co-development of under assistance diaphragm myotrauma and self-inflicted lung injury [15, 16]. An acceptable level of muscle unloading while preserving spontaneous breathing is generally suggested as the most reasonable strategy to avoid complications and achieve a successful weaning from mechanical ventilation [17]. The growing use of ultrasound as a diagnostic tool has made the bedside monitoring of diaphragm function easily performed in an accurate, reproducible, and noninvasive way [18–20]. The assessment of diaphragm function is performed with the evaluation of its excursion, or swing, and the inspiratory thickening. To the best of our knowledge, we are not aware of any study on the effect of acupuncture on diaphragm function, likely both because the ability to assess diaphragm function at the bedside with noninvasive techniques such as ultrasonography is a recent acquisition in western medicine, and because the diaphragm, meant merely as a muscle, is not conceptually relevant to TCM. The aim of this proof-of-concept, prospective, monocentric, pilot trial is to assess the diaphragm function in a group of healthy volunteers undergoing a session of acupuncture aimed at improving the function of the diaphragm, interpreted within the TCM perspective.

## 2. Materials and Methods

### 2.1. Subjects

Healthy volunteers were considered for enrolment. Exclusion criteria were age <18 years, presence of medical condition which could affect diaphragm function (obstructive sleep apnea, lung disease, history of asthma, cardiovascular disease, musculoskeletal abnormalities, or any other serious illness), smoking. A written informed consent was obtained according to Italian regulations.

### 2.2. Protocol

**Figure 1:**
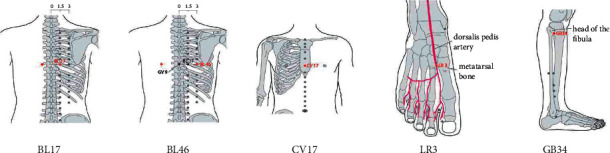
Acupoints selected for diaphragm stimulation protocol.

The entire study was performed in the afternoon in all participants. They were asked to rest in a chair for approximately 20 min after arriving to the laboratory, then seated and, after a period of familiarization with the experimental equipment (mouthpiece and nose clips), they were asked to breathe quietly, with the nose occluded, through a mouthpiece connected to a pneumotachograph. Participants were seated in comfort, breathing quietly during a 15 min period. After a stable respiratory pattern at tidal breathing was attained, the following parameters were determined from an average of 30 s during 5 min: minute ventilation, inspired volume, respiratory rate. At the end of each period patients were asked to perform maximal breaths at vital capacity, and the same parameters were determined. All the clinical and instrumental measurements were taken at baseline, and 20 minutes after application of needles following the acupuncture protocol (Acu) and after sham acupuncture (Sham). Participants allocated to the acupuncture group underwent treatment with needles inserted at the pre-specified acupuncture points, which included BL17 Geshu, BL46 Geguan, CV17 Danzhong, LR3 Tai Chong and GB34 Yanglingquan. All acupoints were localized according to the WHO Standard Acupuncture Locations are summarized in Table 1 and Figure 1. Manipulations of twirling, lifting, and thrusting was performed on all needles for at least 30 s to reach De qi (a compositional sensation including soreness, numbness, distention, and heaviness), which is believed to be an essential component for acupuncture efficacy. Sham acupuncture consisted of a superficial skin penetration (2–3 mm in depth) at non-acupoints, without needle manipulation for De qi. Five prespecified, non-acupoints, away from conventional acupoints or meridians, were stimulated, as shown in Table 1.

Acupuncture and sham acupuncture were randomly performed for each patient, during a 20 min session. Body position was kept constant during the three steps. Stimulation of acupoints was performed with sterile, single-use, disposable, silver-handle, and stainless-steel needles with a traditional pinecone tip for a painless puncture (Boenmed, 0.25 mm × 0.30 mm, Boen Healthcare Co. Ltd, Jiangsu, China).

### 2.3. Measurements

The flow was recorded using a heated, calibrated pneumotachograph (Fleisch No. 3; Fleish, Lausanne, Switzerland) connected to a laptop for subsequent, offline analysis (Colligo, Elekton, Milan, Italy). The Computo software (Elekton, Milan, Italy) was used to display airway flow, volume, and pressure waveforms values.

Ultrasonographic evaluation of the right hemidiaphragm was performed by the same trained operator (MU). Hemodynamic and respiratory parameters were recorded, as well as inspiratory and expiratory diaphragm thickness (Tdi, ei and Tdi, ee, respectively), and diaphragm thickening ratio (TR) during tidal and maximal breathing (indicating inspiratory effort and maximal muscle function, respectively) [18, 19, 21–23]. Images were recorded for a subsequent, computer-assisted quantitative analysis by a trained investigator (MU), unaware of the acupuncture condition.

Diaphragm thickness was assessed in the zone of apposition of the diaphragm to the rib cage. The linear probe was placed above the right 10th rib in the mid-axillary line, as previously described [24]. The inferior border of the costophrenic sinus was identified as the transition from the artefactual representation of the lung to the visualization of the liver. Three subsequent measures were averaged. The diaphragm thickening ratio was calculated as follows:

(1) $$\begin{array}{c} \text{TR}=\frac{\left(\text{end}-\text{inspiratory thickness}-\text{end}-\text{expiratory thickness}\right)}{\text{end}-\text{expiratory thickness}}*100 \end{array}$$

Pulse analysis was performed following CTM bilaterally at both wrists. The pulse was classified as superficial/deep, large/small, long/short, slippery/rough, slow/rapid, strong/soft, fine/weak, and slippery/rough. Tongue diagnosis was also performed on each participant, with observation of the surface of the tongue focused on the shape, fur, and body of the tongue.

Hemodynamic parameters were recorded using a standard multiparametric monitor (HP Monitor M1046A; Hewlett-Packard, Andover, USA); ultrasound was performed using a LogiQ7 device (GE Healthcare, Little Chalfont, UK).

### 2.4. Randomization and Masking

After baseline assessment, participants were randomly assigned in a crossover fashion to the acupuncture or sham acupuncture group. Due to the nature of acupuncture, masking of the acupuncturist was difficult to achieve; participants, ultrasound assessor, and the investigator who performed the statistical analyses were blinded to group assignment. Participant's allocated intervention was not revealed until the statistical analysis was completed.

### 2.5. Statistics

Based on data from healthy volunteers [24], in whom an average thickening ratio of 120 ± 30% is expected with maximal breathing, and hypothesizing that acupuncture may lead to a 30% increase in diaphragm thickening, we calculated that a sample size of 10 subject would allow us to have a power of 80% at an alpha level of 0.05. Data were analyzed using Stata 13.0 (StataCorp, College Station, Texas, USA) for Windows. Normality was assessed by the Shapiro-Francia test. Descriptive results are reported as mean (standard deviation) if normally distributed, or median [25th—75th percentiles] otherwise. The analysis on the variables recorded over the three steps (baseline, sham, acupuncture) was performed by analysis of variance for repeated measurements, with step as a within-participant factor, and the statistical significance of the within-participant factors was corrected with the Greenhouse–Geisser method. Nonparametric variables were analyzed using the Friedman test. Pairwise, post-hoc multiple comparisons were carried out according to the Tukey method. Two-tailed *p* values < 0.05 were considered for statistical significance.

## 3. Results

**Figure 2:**
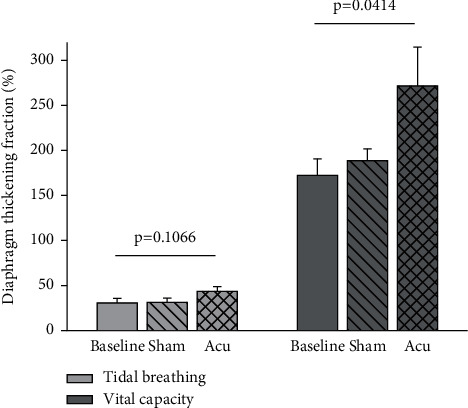
Change in diaphragm thickening fraction in the different steps of the study. Acu: acupuncture group.

Ten volunteers were enrolled: 6 males, 4 females. Body weight was 71 ± 12 kg, height was 173 ± 9 cm, BMI was 21 ± 1.3 kg/m^2^.  Table 2 reports the individual clinical characteristics of the participants enrolled in the study.  Table 3 shows the vital parameters and diaphragm ultrasound results in the different steps of the study. Heart rate, blood pressure, respiratory rate, and SpO_2_ were stable during the whole investigation. During tidal breathing, tidal volume (602 ± 197 vs. 653 ± 195 vs. 671 ± 174 ml, *p*=0.7067), diaphragm displacement, and end-expiratory diaphragm thickness did not change with sham or real acupuncture as compared to baseline. End-inspiratory diaphragm thickness showed a 10%, non-significant increase in the acupuncture group, and thickening ratio was 43.5 ± 16.6 vs. 31.3 ± 14.9 (sham) vs. 30.8 ± 15.3% (baseline), *p*=0.1066. During breaths at vital capacity, the acupuncture group, as compared to both baseline and sham acupuncture, had a trend toward a larger tidal volume (3840 ± 690 vs. 3110 ± 880 vs. 3230 ± 750 ml, *p*=0.1247) and showed a significantly higher thickening ratio (270.6 ± 136.4 vs. 172.4 ± 57.4 vs. 188.6 ± 41.7%, *p*=0.0414).  Figure 2 shows the changes in diaphragm thickening fraction in the different steps of the study both during tidal breathing and when breathing at vital capacity.

## 4. Discussion

The main result of the current pilot, proof-of-concept investigation is that acupuncture significantly influenced diaphragm function in healthy volunteers, improving its inspiratory thickening fraction during breaths at vital capacity, as compared to no or sham acupuncture. During artificial ventilator support, the work of breathing is shared between the patient and the mechanical ventilator. Several indices have been proposed to optimize the level of ventilator assistance with the aim of a best balance between muscle unloading to avoid fatigue and myotrauma and excessive support with the potential result of over assistance and disuse atrophy [16]. Respiratory rate, tidal volume, and other noninvasive parameter derived from the ventilator waveforms have all been used to this extent [21, 22, 25]. However, they generally have shown limited sensitivity and specificity and often require patient cooperation. As outlined above, the growing use of diaphragm ultrasound provided the clinicians with a sensitive, bedside-available, non-invasive tool to monitor diaphragm function during both spontaneous and assisted breathing. During inspiration, the diaphragm contracts and shortens; since its total volume must not change, this leads to an increase in the muscle thickens, and this thickening can be visualized on ultrasound [24]. Diaphragm thickening fraction is the magnitude of the increase in diaphragm thickness during inspiration.

While the displacement is generally used as an index of caudal descent of the diaphragm dome, the inspiratory thickening has shown a stronger relationship with indices of inspiratory effort and was recently shown to be a more sensitive parameter of diaphragm efficiency [20]. As expected, being our case-mix composed by healthy individuals, the overall diaphragm function was well within the limits of normality as reported for spontaneously breathing participants as identified by a displacement of 19.4 ± 11.3 mm at baseline [18]. Notably, neither true nor sham acupuncture significantly modified the diaphragm displacement or the inspiratory thickening during tidal breathing. Indeed, the thickening fraction during tidal breathing showed a trend towards increased values during true acupuncture (43.5 ± 16.6%) vs. sham (31.3 ± 14.9%) and baseline (30.8 ± 15.3%), *p*=0.1066. During maximal breathing, however, true acupuncture led to a significantly improved inspiratory thickening of the diaphragm (270.6 ± 136.4%) vs. sham (188.6 ± 41.7%) and baseline (172.4 ± 57.4%), *p*=0.0414. This was associated to increased, although not statistically significant, inspired volume, and diaphragm displacement (Table 3).

When the diaphragm is activated during tidal breathing, it shortens and hence thickens. Since thickening fraction is related to the degree of diaphragmatic shortening during inspiration, it correlates with the changes in lung volume [20], inspiratory pressure development [23], and work of breathing [18].

The apparently counterintuitive dissociation between an increased thickening fraction and the lack for a statistically significant difference in inspired volume and diaphragm excursion may lie in the limited sample size (and hence in the possibility of a type II error) of our study. Nevertheless, a dissociation between diaphragm excursion and inspiratory thickening has been recently described and can have some degree of pathophysiological plausibility (the thickening fraction being related to the muscular function, while the excursion being associated with the change in the lung volume) [26].

We hypothesize that the acupuncture stimulation performed in the current investigation might have improved on the one side the energy associated with “wood,” while on the other it might have improved the communication between the “upper” and the “lower,” thanks to the action played on the diaphragm acupoints. Several different acupoints have been associated with the diaphragm in different ways.  Figure 2 summarizes the acupoints selected for the current investigation. The first acupoint is BL 17-gěshū. In the Nánjīng, it is described as the “meeting point of blood Gěshū.” This point is known as master of blood, as the heart which lays above it produces the blood, while the liver which lays beneath it drains it. In fact, it represents the Shu point of the diaphragm, and the Hui point of Xue, and it is commonly used to move the blood and the Qi. It is located 1.5 cun lateral to the median vertebral line, at the level of the VII dorsal vertebra. Within TCM, its effects are to feed the blood and make it flow, to tonify Qi and Xue, to remove the obstructions of the diaphragm and release the organ, to subdue the stomach Qi Ni, to open the chest, to purify the heat, and to calm the Shen. In western medicine, it is used to treat anemia, fatigue, hiccups, dysphagia, vomit, cough, asthma, and dyspnea [7]. This point is often used in tonification (lifting and thrusting with the needle) or in moxa (heating the acupoint with burning small cones of dried leaves), it feeds the blood of any Zang, especially if it is associated with the Mu point which corresponds to the affected organ. Moreover, it moves the Qi in the diaphragm and in the chest, and it is indicated for feelings of fullness of the chest or epigastric distension with regurgitation, nausea or vomiting. A point immediately lateral to BL17 is BL46-Gěguān. This point represents the opening point of the diaphragm, and the opening of the “steel cauldron.” It is located 3 cun lateral to the median vertebral line, at the same level of the VII dorsal vertebra. It is used in TCM for stasis in the chest and stomach Qi Ni, as it scatters the distension of the chest, it opens the diaphragm, it favors the descent of Qi of the stomach. In western medicine, it is indicated for dysphagia, regurgitation, vomiting, and stiffness of the head, as well as diaphragm pathology and symptoms of Qi Ni [7]. CV17-Danzhong, is located in the anterior part of the body. This point represents the middle of the chest, where middle means in the center but also central, impartial, moderate strength, and supreme domination of the chaos [10]. It is the Mu point or Master of the Heart and of the superior Jao, Hui point of the Qi, and the reunion point of the great longitudinal Luo meridian triple warmer. This point is located on the midline level within the 4th intercostal space midway between the nipples. It is used in TCM for its effect of regulating Qi and the treating the blood stasis, it clears the lung when a lung Yin deficiency occurs and transforms phlegm, loosens the chest, and disinhibits the diaphragm. In western medicine, it is used for dyspnea, hiccups, chest pain, wheezing and shortness of breath, and heart palpitations with sorrow and fear.

In a case report, acupuncture of GV 8 (JinSuo), GV 6 (JiZhong), GV 7 (ZhongShu), GV 20 (BaiHui), LI6 (PianLi), KI16 (Huangshu), CV4 (GuanYuan), CV11 (JianLi), ST36 (Zusanli), SP6 (SanYinJiao), and LV3 (TaiChong) proved an effective treatment for diaphragm muscle pain in a young male who experienced diaphragm damage after peptic ulcer surgery [5]. Recently, a systematic review was performed to investigate the potential rehabilitation effects of acupuncture on diaphragm dysfunction in patients with chronic obstructive pulmonary disease (COPD) [27]. The authors retrieved a total of 9 papers: 7 studies added acupuncture to standard treatment for patients with diaphragm dysfunction in COPD and found statistically significant changes in the maximum inspiratory pressure and the use of accessory inspiratory muscles. The remaining studies shown that acupuncture, combined with other TCM methods in the rehabilitation of COPD, improved the diaphragm strength. Notably, none of these papers used ultrasound as a diagnostic tool for diaphragm function.

To the best of our knowledge, this is the first study to investigate diaphragm function, as assessed by ultrasound, in healthy volunteers. Given the pivotal role that diaphragm function plays in critically ill patients undergoing assisted mechanical ventilation, we believe that the current investigation might form the proof-of-concept basis for further, clinical studies on the impact of acupuncture on diaphragm function. Moreover, it is becoming increasingly apparent that diaphragm dysfunction is present in a high percentage of critically ill patients and is associated with increased morbidity and mortality [28]. In these patients, diaphragm weakness is thought to develop from disuse secondary to ventilator-induced diaphragm inactivity and because of the effects of systemic inflammation, including sepsis. This form of critical illness-acquired diaphragm dysfunction impairs the ability of the respiratory pump to compensate for an increased respiratory workload due to lung injury and fluid overload, leading to sustained respiratory failure and death. On top of that, no defined treatment has been identified for this condition [29], and we speculate that these results, if replicated in the clinical setting, might represent a possible treatment option. With this regard, our preliminary results may be applied in those patients diagnosed with diaphragmatic disfunction, with the aim to improve the inspiratory function of the muscle while limiting the above-mentioned adverse effects.

Our study has limitations that need to be discussed. First, the sample size is limited; however, it is of similar magnitude to other physiological investigations [16]. Second, respiratory parameters and diaphragm ultrasound has been performed in the semirecumbent position; the results cannot be directly extrapolated to participants lying in different positions. However, the semirecumbent position is the most common position in participants undergoing weaning from ventilator assistance. Third, given the different role of the diaphragm in TCM as compared to western medicine, the diaphragm acupuncture stimulation protocol was designed by the authors of the current investigation, based on clinical reasoning, as we could not draw from an established tradition. Last, we did not use gold-standard indices of inspiratory effort such as the measurement of esophageal and trans-diaphragmatic pressures. The latter are in fact invasive measures, and ultrasound has consistently shown a strong correlation with such indices.

In conclusion, in healthy volunteers, acupuncture led to a significant improvement in diaphragm inspiratory thickening ratio during breaths at vital capacity. As preliminary results, these must be confirmed in larger clinical studies, and subsequently in critically ill patients in which diaphragmatic dysfunction has been diagnosed, with the final aim to potentially reduce the length of mechanical ventilation and of ICU stay.

## Figures and Tables

**Table 1:** List and location of acupoints and non-acupoints during true and sham acupuncture.

Acupoints	Location
Geshu (BL17)	1.5 cun lateral to the median vertebral line, at the level of the VII dorsal vertebra
Geguan (BL46)	3 cun lateral to the median vertebral line, at the level of the VII dorsal vertebra
Danzhong (CV17)	Midline level within the 4th intercostal space midway between the nipples
Tai chong (LR3)	On the dorsum of the foot, in the depression proximal to the 1st metatarsal space
Yanglingquan (GB34)	1 cun anterior inferior to the head of the fibula below the lateral part of the knee

Non-acupoints	Location
Non-acupoint 1	In the middle of touwei (ST8) and yuyao (EX-HN4) points
Non-acupoint 2	2 cun above the anterior superior iliac spine
Non-acupoint 3	2 cun below the umbilicus, and 1 cun lateral to the anterior midline.
Non-acupoint 4	In the middle of the humerus medial epicondyle and the styloid process of ulna
Non-acupoint 5	3 cun below yanglingquan (GB34), between the gallbladder and bladder meridian

1 cun (≈20 mm) is defined as the width of the interphalangeal joint of subject's thumb.

**Table 2:** Clinical characteristics of the enrolled participants.

Subj	Age (years)	Sex	Weight (kg)	Height (cm)	BMI (kg/m^2^)	Pulse	Condition
1	28	M	72	185	21, 0	Deep, regular, slow, soft, weak, small, rough	Non-smoker
2	42	M	68	177	21, 7	Superficial, regular, rapid, strong, fine, large, slippery	Smoker
3	27	M	70	175	22, 9	Deep, regular, slow, soft, fine, large, slippery	Smoker
4	32	M	81	180	25, 0	Superficial, regular, rapid, soft, weak, small, rough	Smoker
5	50	M	78	172	26, 4	Deep, regular, slow, soft, weak, small, rough	Non-smoker
6	26	F	58	167	20, 8	Deep, regular, slow, strong, fine, large, slippery	Non-smoker
7	29	F	55	170	19, 0	Deep, regular, slow, soft, weak, small, slippery	Non-smoker
8	26	F	50	162	19, 1	Deep, regular, slow, soft, weak, small, slippery	Non-smoker
9	27	M	75	175	24, 5	Deep, regular, slow, strong, fine, large, rough	Non-smoker
10	25	F	62	179	19, 4	Deep, regular, slow, soft, weak, small, slippery	Non-smoker

**Table 3:** Vital parameters and diaphragm ultrasound results in the different steps of the study.

	Baseline	Sham	Acu	*p*
Respiratory rate (1/min)	16 ± 3	14 ± 4	16 ± 4	0.3436
SpO_2_ (%)	98.6 ± 1.8	99.2 ± 1.4	99.4 ± 1.2	0.4927
Heart rate (1/min)	66.8 ± 10.8	68.1 ± 11.3	68.4 ± 11.7	0.9454
Systolic blood pressure (mmHg)	123.8 ± 11.3	122.7 ± 14.0	123.0 ± 15.2	0.9829
Diastolic blood pressure (mmHg)	76.9 ± 8.3	78.1 ± 6.1	78.9 ± 8.9	0.8514

Tidal breathing				
Inspired volume (mL)	602 ± 197	653 ± 195	671 ± 174	0.7067
Diaphragm displacement (mm)	19.4 ± 11.3	21.2 ± 12.2	18.6 ± 9.6	0.8701
End-inspiratory diaphragm thickness (mm)	1.55 ± 0.27	1.55 ± 0.23	1.56 ± 0.32	0.9968
End-expiratory diaphragm thickness (mm)	2.02 ± 0.29	2.02 ± 0.30	2.20 ± 0.34	0.3119
Diaphragm thickening fraction (%)	30.8 ± 15.3	31.3 ± 14.9	43.5 ± 16.6	0.1066

Vital capacity				
Inspired volume (ml)	3110 ± 880	3230 ± 750	3840 ± 690	0.1247
Diaphragm displacement (mm)	56.5 ± 13.8	55.9 ± 17.3	64.7 ± 18.5	0.2811
End-inspiratory diaphragm thickness (mm)	1.66 ± 0.29	1.68 ± 0.32	1.52 ± 0.27	0.4595
End-expiratory diaphragm thickness (mm)	4.47 ± 1.01	4.89 ± 1.51	5.48 ± 1.72	0.3088
Diaphragm thickening fraction (%)	172.4 ± 57.4	188.6 ± 41.7	270.6 ± 136.4	0.0414

## Data Availability

The data used to support the findings of this study are available from the corresponding author upon request.

## References

[1] Pickering M., Jones J. F. X. (2002). The diaphragm: two physiological muscles in one. *Journal of Anatomy*.

[2] Hodges P. W., Heijnen I., Gandevia S. C. (2001). Postural activity of the diaphragm is reduced in humans when respiratory demand increases. *The Journal of Physiology*.

[3] Ferraro F. V., Gavin J. P., Wainwright T., McConnell A. (2019). The effects of 8 weeks of inspiratory muscle training on the balance of healthy older adults: a randomized, double‐blind, placebo‐controlled study. *Physiological Reports*.

[4] Bernapel C., Charles G. (1996). *Taiji Quan—Pratique et Enseignement des Huit Portes et Treize Postures (Ba men shi san shi)*.

[5] Kong Y. C. (2011). Huangdi Neijing: a synopsis with commentaries. *China Review International*.

[6] Longhurst J. C. (2010). Defining meridians: a modern basis of understanding. *Journal of Acupuncture and Meridian Studies*.

[7] Wallden M. (2017). The diaphragm—more than an inspired design. *Journal of Bodywork and Movement Therapies*.

[8] Deadman P., Al-Khafaji M., Baker K. (1998). *A Manual of Acupuncture*.

[9] McCool F. D., Tzelepis G. E. (2012). Dysfunction of the diaphragm. *New England Journal of Medicine*.

[10] Lone N. I., Walsh T. S. (2011). Prolonged mechanical ventilation in critically ill patients: epidemiology, outcomes and modelling the potential cost consequences of establishing a regional weaning unit. *Critical Care*.

[11] Powers S. K., Shanely R. A., Coombes J. S. (2002). Mechanical ventilation results in progressive contractile dysfunction in the diaphragm. *Journal of Applied Physiology*.

[12] Sassoon C. S., Caiozzo V. J., Manka A., Sieck G. C. (2002). Altered diaphragm contractile properties with controlled mechanical ventilation. *Journal of Applied Physiology*.

[13] Shanely R. A., Coombes J. S., Zergeroglu A. M., Webb A. I., Powers S. K. (2003). Short-duration mechanical ventilation enhances diaphragmatic fatigue resistance but impairs force production. *Chest*.

[14] Vassilakopoulos T., Petrof B. J. (2004). Ventilator-induced diaphragmatic dysfunction. *American Journal of Respiratory and Critical Care Medicine*.

[15] Bertoni M., Spadaro S., Goligher E. C. (2020). Monitoring patient respiratory effort during mechanical ventilation: lung and diaphragm-protective ventilation. *Critical Care*.

[16] Goligher E. C., Dres M., Patel B. K. (2020). Lung-and diaphragm-protective ventilation. *American Journal of Respiratory and Critical Care Medicine*.

[17] Goligher E. C., Ferguson N. D., Brochard L. J. (2016). Clinical challenges in mechanical ventilation. *The Lancet*.

[18] Boussuges A., Gole Y., Blanc P. (2009). Diaphragmatic motion studied by M-mode. *Chest*.

[19] Kim W. Y., Suh H. J., Hong S.-B., Koh Y., Lim C.-M. (2011). Diaphragm dysfunction assessed by ultrasonography: influence on weaning from mechanical ventilation. *Critical Care Medicine*.

[20] Wait J. L., Johnson R. L. (1997). Patterns of shortening and thickening of the human diaphragm. *Journal of Applied Physiology*.

[21] DiNino E., Gartman E. J., Sethi J. M., McCool F. D. (2014). Diaphragm ultrasound as a predictor of successful extubation from mechanical ventilation. *Thorax*.

[22] Umbrello M., Formenti P., Longhi D. (2015). Diaphragm ultrasound as indicator of respiratory effort in critically ill patients undergoing assisted mechanical ventilation: a pilot clinical study. *Critical Care*.

[23] Ueki J., De Bruin P. F., Pride N. B. (1995). In vivo assessment of diaphragm contraction by ultrasound in normal subjects. *Thorax*.

[24] Umbrello M., Formenti P. (2016). Ultrasonographic assessment of diaphragm function in critically ill subjects. *Respiratory Care*.

[25] Kantarci F., Mihmanli I., Demirel M. K. (2004). Normal diaphragmatic motion and the effects of body composition. *Journal of Ultrasound in Medicine*.

[26] Kracht J., Ogna A., Fayssoil A. (2020). Dissociation between reduced diaphragm inspiratory motion and normal diaphragm thickening in acute chronic pulmonary obstructive disease exacerbation: a case report. *Medicine*.

[27] Liu Q., Duan H., Lian A., Zhuang M., Zhao X., Liu X. (2021). Rehabilitation effects of acupuncture on the diaphragm dysfunction in chronic obstructive pulmonary disease: a systematic review. *International Journal of Chronic Obstructive Pulmonary Disease*.

[28] Dres M., Goligher E. C., Heunks L. M. A., Brochard L. J. (2017). Critical illness-associated diaphragm weakness. *Intensive Care Medicine*.

[29] Dubé B. P., Dres M. (2016). Diaphragm dysfunction: diagnostic approaches and management strategies. *Journal of Clinical Medicine*.

